# Comparison of non-crystalline silica nanoparticles in IL-1β release from macrophages

**DOI:** 10.1186/1743-8977-9-32

**Published:** 2012-08-10

**Authors:** Wiggo J Sandberg, Marit Låg, Jørn A Holme, Bernd Friede, Maurizio Gualtieri, Marcin Kruszewski, Per E Schwarze, Tonje Skuland, Magne Refsnes

**Affiliations:** 1 Norwegian Institute of Public Health, Division of Environmental Medicine, P.O. Box 4404, Nydalen, Oslo, N-0403, Norway; 2 Elkem AS, Silicon Materials, P.O. Box 8126, Vaagsbygd, Kristiansand, 4675, Norway; 3 Present address: Research Centre POLARIS, Department of Environmental Science, University Milano-Bicocca, Piazza della Scienza 1, Milan, 20126, Italy; 4 Institute of Nuclear Chemistry & Technology, Warsaw, Poland; 5 Independent Laboratory of Molecular Biology, Institute of Rural H, Jaczewskiego 2, Lublin, 20-950, Poland

**Keywords:** Non-crystalline and crystalline silica particles, Particle size, Macrophages, Inflammation, IL-1β, NALP3 inflammasome, Particle uptake, Phagosomal destabilization

## Abstract

**Background:**

Respirable crystalline silica (silicon dioxide; SiO_2_, quartz) particles are known to induce chronic inflammation and lung disease upon long-term inhalation, whereas non-crystalline (amorphous) SiO_2_ particles in the submicrometre range are regarded as less harmful. Several reports have demonstrated that crystalline, but also non-crystalline silica particles induce IL-1β release from macrophages via the NALP3-inflammasome complex (caspase-1, ASC and NALP3) in the presence of lipopolysaccharide (LPS) from bacteria. Our aim was to study the potential of different non-crystalline SiO_2_ particles from the nano- to submicro-sized range to activate IL-1β responses in LPS-primed RAW264.7 macrophages and primary rat lung macrophages. The role of the NALP3-inflammasome and up-stream mechanisms was further explored in RAW264.7 cells.

**Results:**

In the present study, we have shown that 6 h exposure to non-crystalline SiO_2_ particles in nano- (SiNPs, 5–20 nm, 50 nm) and submicro-sizes induced strong IL-1β responses in LPS-primed mouse macrophages (RAW264.7) and primary rat lung macrophages. The primary lung macrophages were more sensitive to Si-exposure than the RAW-macrophages, and responded more strongly. In the lung macrophages, crystalline silica (MinUsil 5) induced IL-1β release more potently than the non-crystalline Si50 and Si500, when adjusted to surface area. This difference was much less pronounced versus fumed SiNPs. The caspase-1 inhibitor zYVAD and RNA silencing of the NALP3 receptor reduced the particle-induced IL-1β release in the RAW264.7 macrophages. Furthermore, inhibitors of phagocytosis, endosomal acidification, and cathepsin B activity reduced the IL-1β responses to the different particles to a similar extent.

**Conclusions:**

In conclusion, non-crystalline silica particles in the nano- and submicro-size ranges seemed to induce IL-1β release from LPS-primed RAW264.7 macrophages via similar mechanisms as crystalline silica, involving particle uptake, phagosomal leakage and activation of the NALP3 inflammasome. Notably, rat primary lung macrophages were more sensitive with respect to silica-induced IL-1β release. The differential response patterns obtained suggest that silica-induced IL-1β responses not only depend on the particle surface area, but on factors and/or mechanisms such as particle reactivity or particle uptake. These findings may suggest that bacterial infection via LPS may augment acute inflammatory effects of non-crystalline as well as crystalline silica particles.

## Background

The general population is exposed to crystalline silica (silicon dioxide; SiO_2_) particles, abundant in nature as quartz and other minerals. Airborne quartz particles are also encountered in numerous occupations, including mining and construction work. Chronic exposure to respirable quartz particles is associated with ongoing cell injury, fibrosis (silicosis) and lung cancer [1-3]. Non-crystalline silica (amorphous) particles are generally regarded to be safer, with no or less chronic effects [4-6]. Thus, non-crystalline silica particles are now increasingly used for various applications including construction work, medical diagnosis, cancer therapy and drug delivery, and are added to cosmetics and food. However, the increasing use of various forms of non-crystalline silica particles, and in particular the nano-sized, requires more thorough examination of their possible health effects.

Scavenging of quartz particles by resident macrophages leads to direct release of inflammatory cytokines, which seems to be crucial in the development of silicosis [1,7]. Notably, a persistent overproduction of the pro-inflammatory cytokine IL-1β has been linked to silicosis [8]. Reduction of experimentally-induced silicosis has been observed upon treatment with IL-1 receptor antagonists as well as in IL-1β knockout mice, confirming IL-1β as a key inflammatory regulator [9,10]. Furthermore, other studies have shown that crystalline silica particles (quartz) can trigger the release of IL-1β from macrophages [11].

Extensive research in recent years has revealed that the IL-1β response is regulated by two separate mechanisms. First activation of toll-like receptors (TLRs) is leading to increased levels of pro-IL-1β. The next step may involve the intracellular pattern recognition receptor NALP3, resulting in maturation and release of IL-1β. TLRs are often activated by ligation of pathogen-associated molecular pattern (PAMP) and danger associated molecular pattern (DAMP) molecules. This triggers a cellular signaling cascade, which increases transcription of the pro-IL-1β gene via the transcription factor NFκ-B. The NALP3 is a multi-ligand sensing protein that recognizes a variety of host-derived danger signals, including DAMPs. Upon ligation, the NALP3 binds the adaptor protein ASC and pro-caspase-1 in the inflammasome multi-protein complex. The inflammasome assembly will spontaneously activate caspase-1, which cleaves pro-IL-1β and causes a release of IL-1β [12-14]. Partly based on the successful treatment with IL-1β receptor antagonists, the NALP3 inflammasome is suggested to have an important pathogenic role in several inflammatory diseases including gout, type 2 diabetes and cardiovascular disease [15-21].

Several studies have focused on the crystalline structure of particles as a crucial factor for triggering the inflammasome response. Accordingly, biologically relevant crystals, such as monosodium urate (MSU), have been suggested to be important for the NALP3/IL-1β-driven inflammation in gout disease [22,23]. Cholesterol crystals seem to contribute to atherogenesis via inflammasome activation [24]. It has also been reported that the crystalline silica particles (quartz) activate the NALP3 inflammasome, possibly via particle uptake, rupture of lysosomal membranes and subsequent release of cathepsin B [14,25]. The quartz-induced NALP3 activation and subsequent IL-1β release has recently been more directly linked to the development of silicosis upon chronic exposure [26].

A critical question is to what extent the inflammasome activation by silica particles requires the crystalline structure, or whether non-crystalline (amorphous) silica particles of different sizes also may induce inflammasome responses. Several studies have reported that non-crystalline silica particles may induce inflammasome activation, but the results differ depending on the particle size and the cell types examined [27-29]. In the present *in vitro* study we have investigated the potential of different non-crystalline silica particles to induce IL-1β release from LPS-primed RAW264.7 macrophage as well as primary rat lung macrophages. The macrophages were exposed to such silica particles in the nano- and submicro-ranges (50 and 500 nm nominal size) and of two types of poly-disperse non-crystalline silica particles of industrial origin (fumed silica, fused silica). To compare with crystalline silica micrometer-sized quartz (MinUsil 5) was included. The mechanisms behind the IL-1β release in the RAW264.7 macrophages were investigated, including the role of particle phagocytosis, lysosomal membrane stability, release of lysosomal proteases and NALP3 activation.

## Results

### Characterization of the particles

**Figure 1:**
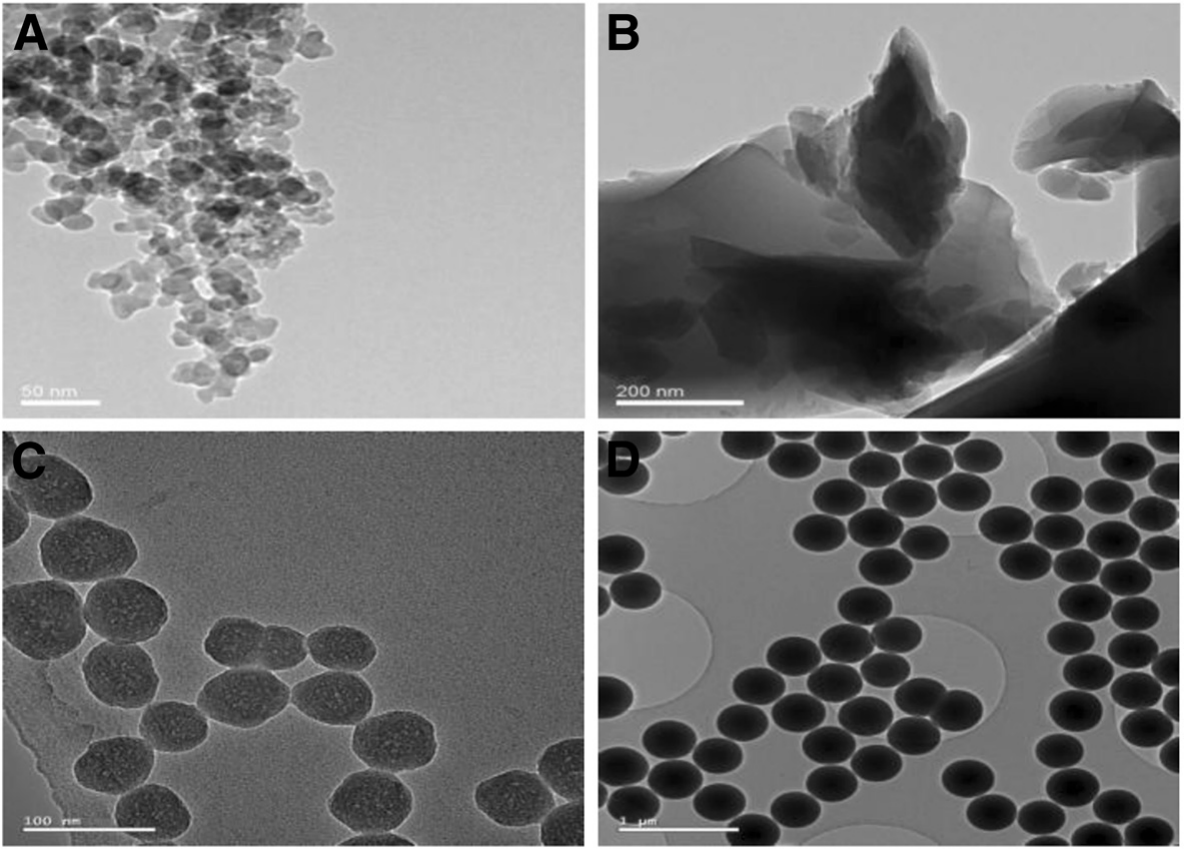
**Morphology of various non-crystalline silica particles.** TEM-micrographs of the particles; using Philips CM30; FEG, 300 kV, are presented. **A**) Fumed silica (Aerosil 200, scale bar: 50 nm) **B**) Fused silica (Suprasil, scale bar 200 nm) before wet sedimentation **C**) 50 nm monodisperse silica (Si50, scale bar: 100 nm) **D**) 500 nm monodisperse silica (Si500, scale bar: 1 μm).

Non-crystalline silica particles in the nanometer- and submicro-sizes, expected to be both mono-disperse and poly-disperse (see Materials and Methods), were characterized with regard to morphology size (TEM, DLS), BET surface area and surface charge (ζ-potentials). In Figure 1 TEM micrographs are shown and a typical aggregate of fumed silica is presented in Figure 1A. The primary particles are approximately 20 nm in size; and they are sintered together thereby forming an open porous network. In Figure 1B the angular morphology of cleaved fused silica particles is clearly illustrated. These micrographs represent the particles before wet sedimentation. Sinterbridges, as visible in the TEM micrographs of the fused silica particles, are artefacts of the wet sample preparation. The quasi-spherical, mono-disperse Si50 particles and the perfectly spherical, mono-disperse Si500 particles are depicted in Figure 1C and 1D, respectively. In contrast to fumed silica, the primary spherical particles form loose agglomerates due to van-der-Waals forces.

Table 1 shows the nominal sizes given for the particles as well as sizes measured in our TEM-instrument. In addition the surface areas by BET-analysis and the ζ-potentials in water are included. TEM investigations revealed that the particle size of the Si50 sample was slightly larger (64 nm) than its nominal size. The particle size of the Si500 sample was 370 nm. Our own measurements are also presented for the fumed and fused silica particles. The BET surface area of fumed silica particles was found to be the largest with 1880 cm^2^/mg. The surface area of fused silica was nearly 1/100 part of this. The surface area of Si50 was approximately a third of the fumed silica particles, and 7-fold higher than their Si500 counterpart. Notably, both the presented values for the TEM measurements and the surface area for the fused silica particles are before wet sedimentation. With respect to the ζ-potentials in water, the particles showed relatively similar values, ranging from −23.9 to −37.2, with the fumed particles as least negative and Si500 as most negative.

**Table 1:** Characteristics of non-crystalline silica particles

**Silica particles**	**TEM (own measurements)**	**Surface area (cm**^2^**/mg)**	**ζ-potential (mV) (in water)**
Silica (50 nm, Si50)	64 ± 7.7 nm	650	−29,8
Silica (500 nm, Si500)	369 ±19.7 nm	90	−37,2
Fumed silica (Aerosil)	~20 nm	1880	−23,9
Fused silica (Suprasil)	^*^500 nm −10 μm	^*^23	^**^-34,4

The characteristics (TEM; surface area, and ζ-potentials) of two different mono-disperse non-crystalline (amorphous) silica particles with different nominal size, and two different poly-disperse amorphous silica particles are presented. The TEM, ζ-potentials (in water), and the particle surface areas were measured as described in Material and Methods. ^*^ The TEM and the surface area of the fused particles were measured before wet sedimentation and include larger particles. ^**^The ζ-potential was measured for the fused particles after wet sedimentation.

Table 2 summarizes the hydrodynamic size by DLS of all the particles dispersed in sterile water with and without sonication and addition of BSA and PBS (stock solution), and after dispersion of the sonicated particles in culture media plus serum, both instantly and after 2 h. In the Additional file 1 the DLS distribution curves are given for Si50 and Si500 (Additional file 1: Figure S1) and fumed and fused silica particles (Additional file 1: Figure S2). The DLS-size of the Si50 in water was approximately of the same size as measured by TEM. After dispersion in a stock solution the DLS-sizes were moderately larger, and these sizes were kept after dispersion in culture medium (DMEM+10% FCS), both instantly and after 2 h. For the Si500, the DLS-size in water was somewhat larger than the measured TEM-size, and was slightly increased in the stock solution and in culture media. For the fumed silica particles a more complex particle size distribution was observed. Dispersed in sterile water these particles showed a bimodal pattern with a peak at 122 nm and one peak at 458 nm (mean intensities), which is due to the aggregation and agglomeration characteristics, respectively. Upon sonication and BSA/PBS supplementation a major peak was observed with a size of 220 nm. Upon dispersion in culture medium, the peak shifted slightly to 295 at 0 h and 255 at 2 h. The fused silica particles, using the smallest fraction after wet sedimentation, displayed a mono-disperse pattern in sterile water with a sharp peak at 342 nm, and somewhat larger (396 nm) after sonication and BSA/PBS supplementation. For all the particles, peaks in the lower size range (7–30 nm) might be due to proteins or other artefacts and the small peaks in the large size area (approximately 5000 nm) might represent agglomeration of particles.

**Table 2:** Size distribution of different non-crystalline silica particles as measured by DLS-measurements

**Silica particles**	**In water**		**In stock-solution(+/-)**		**In medium**		**In medium after 2h**	
	**Fraction 1**	**Fraction 2**	**Fraction 1**	**Fraction 2**	**Fraction 1**	**Fraction 2**	**Fraction 1**	**Fraction 2**
Silica (50 nm,Si50)	68.5		91		91		91	
Silica 500 (500 nm, Si500)	458		531		531		531	
Fumed silica (Aerosil)	122	458	220	1700	295		255	
Fused silica (Suprasil)	342		396		342	5560	342	5560

**Figure 2:**
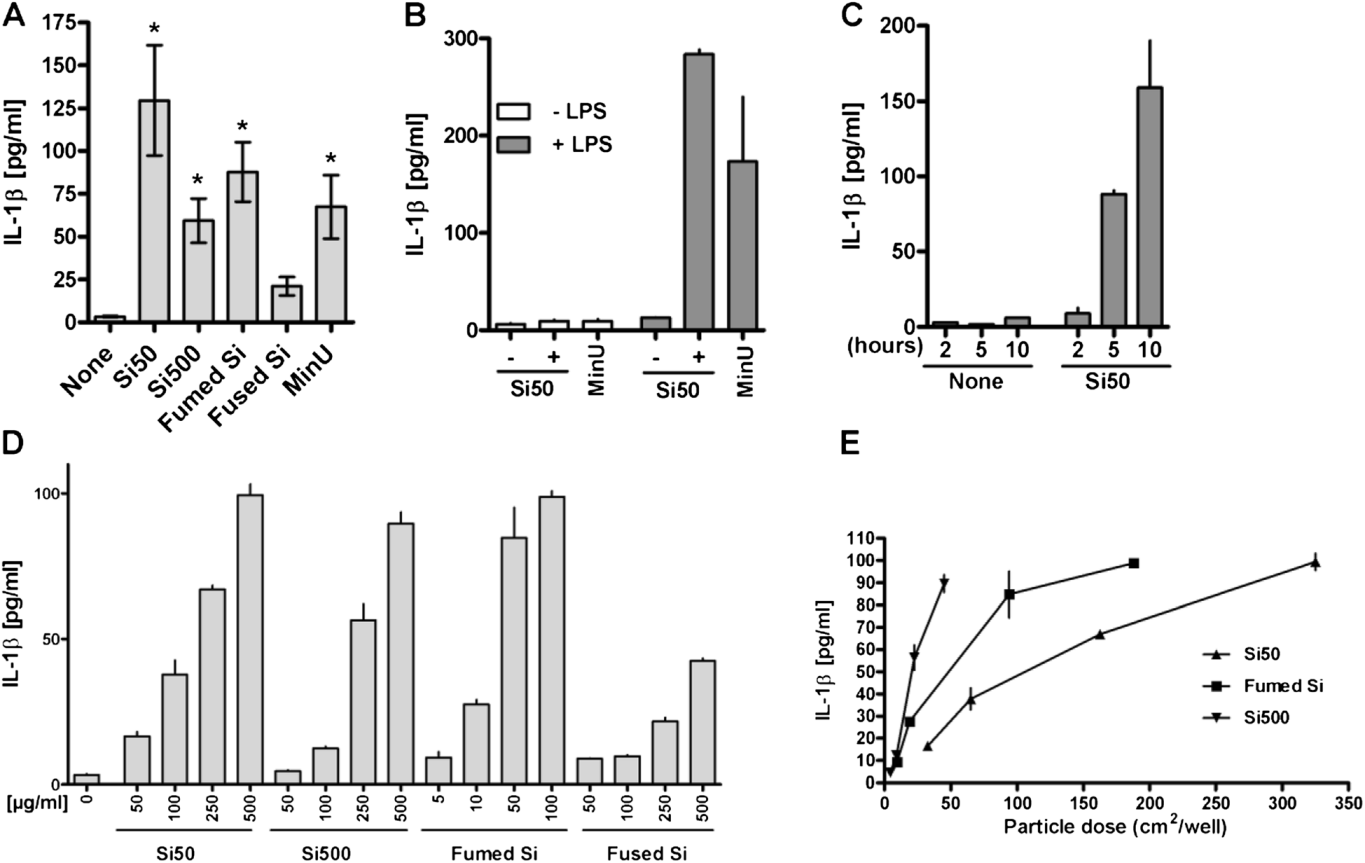
**IL-1β release induced by non-crystalline silica particles in mouse RAW264.7 macrophages.** The cells were primed for 3 h with and without LPS (25 ng/ml) and subsequent exposed to different non-crystalline silica particles and MinUsil 5 (MinU) for 6 h **A**) Effects of Si50, Si500, fumed and fused silicas, besides MinU (200 μg/ml) in LPS-primed cells **B**) Effects with or without LPS and subsequent exposure to Si50 (200 μg/ml) and MinU (200 μg/ml) **C**) Time-dependent effect (2–10 h) by Si50 (200 μg/ml) on IL-1β release in LPS-primed cells **D**) Concentration (mass/ml)-response relationships for Si50, Si500, fumed silica (fumed Si) and fused silica (fused Si) on IL-1β release in LPS-primed cells **E**) IL-1β responses in relation to particle surface areas in LPS-primed cells. The results for Si50, Si500 and fumed silica particles are presented for surface areas. Fused silica particles after wet sedimentation were used. The IL-1β levels were determined by ELISA. In **A**) the variability of the results are presented by the mean ± SD, including data from 3 to 5 experiments. * = p < 0.05 versus control, using Kruskal-Wallis test with Dunn’s Multiple Comparison test as a post-test. In **B-E** representative experiments are given, all performed in duplicates as indicated.

The table presents the sizes for the intensity of the non-crystalline silica particle peaks, as shown in addition file 1, Figure 1 and 2.

### Non-crystalline silica particles induced IL-1β release from LPS-primed RAW264.7 cells

RAW264.7 macrophage cultures were primed with LPS (25 ng/ml, 3 h) before exposure to different silica particles for 6 h (Figure 2). Although significant IL-1β responses were induced by all particles, with exception of fused particles, large variations in the IL-1β responses after exposure to particles could be seen, as illustrated in Figure 2A, showing the effect of a high concentration (200 μg/ml) of Si50; Si500, fumed and fused silica, and MinUsil 5 in LPS-primed cells. However, the relative particle responses within each experiment were similar. Thus, in order to remove this unwanted inter-experimental variation, typical experiments are presented. Figure 2B shows the IL-1β responses to Si50 and MinUsil 5 with and without LPS pre-treatment. Without LPS negligible responses were observed, whereas the responses were strikingly augmented in LPS-primed cells. The response to Si50 was at least of the same magnitude as the response to the same concentration of MinUsil 5. In time-response experiments with LPS-primed cells, using Si50 (200 μg/ml) (Figure 2C), no or only a slight IL-1β response was detected at 2 h, whereas at 5 h a marked response was observed. The response lasted for at least 10 h. In Figure 2D, the concentration-response relationships for Si50-, Si500-, fumed- and fused silica particles with respect to IL-1β responses in LPS-primed cells, as related to mass, are presented. The nano-sized SiNPs showed a leftward shift of the concentration-response curve compared to the larger non-crystalline silica particles. The Si50 induced an effect at 50 μg/ml or higher, whereas Si500 elicited an effect at 100 μg/ml or higher. The responses to Si50 and Si500 increased progressively up to 500 μg/ml. Also the fumed and fused particles both induced IL-1β release in a concentration-dependent manner. The response to the fumed particles was most marked, with a distinct leftward shift compared to the other particles and a distinct response at a concentration as low as 5 μg/ml. The fused silica particles showed a response from 50 μg/ml. In Figure 2E, the IL-1β responses of Si50, Si500 and fumed silica in relation to particle surface area were compared. Si500 seemed to be more potent than Si50, as Si500 reached a response of approximately 90 pg/ml at a surface area of 40 cm^2^/well, and the Si50 reached the same response at around 300 cm^2^/well. The fumed silica particles were more potent than the Si50, with an approximate 3-fold leftward shift. For the fused silica particles the relationship between IL-1β response and surface area is not presented, as the particle surface area of these particles was only measured before wet sedimentation. The cytotoxicity, as assessed by LDH release, was also examined for all these particles. In general, at 100–200 μg/ml the LDH-increase after 6 h exposure was less than 2–2.5 fold with the fumed and fused silica as most and least potent, respectively (data not shown), showing that the measured IL-1β release not was due to unspecific toxic effects.

### IL-1β release from primary lung rat macrophages after exposure to silica particles

**Figure 3:**
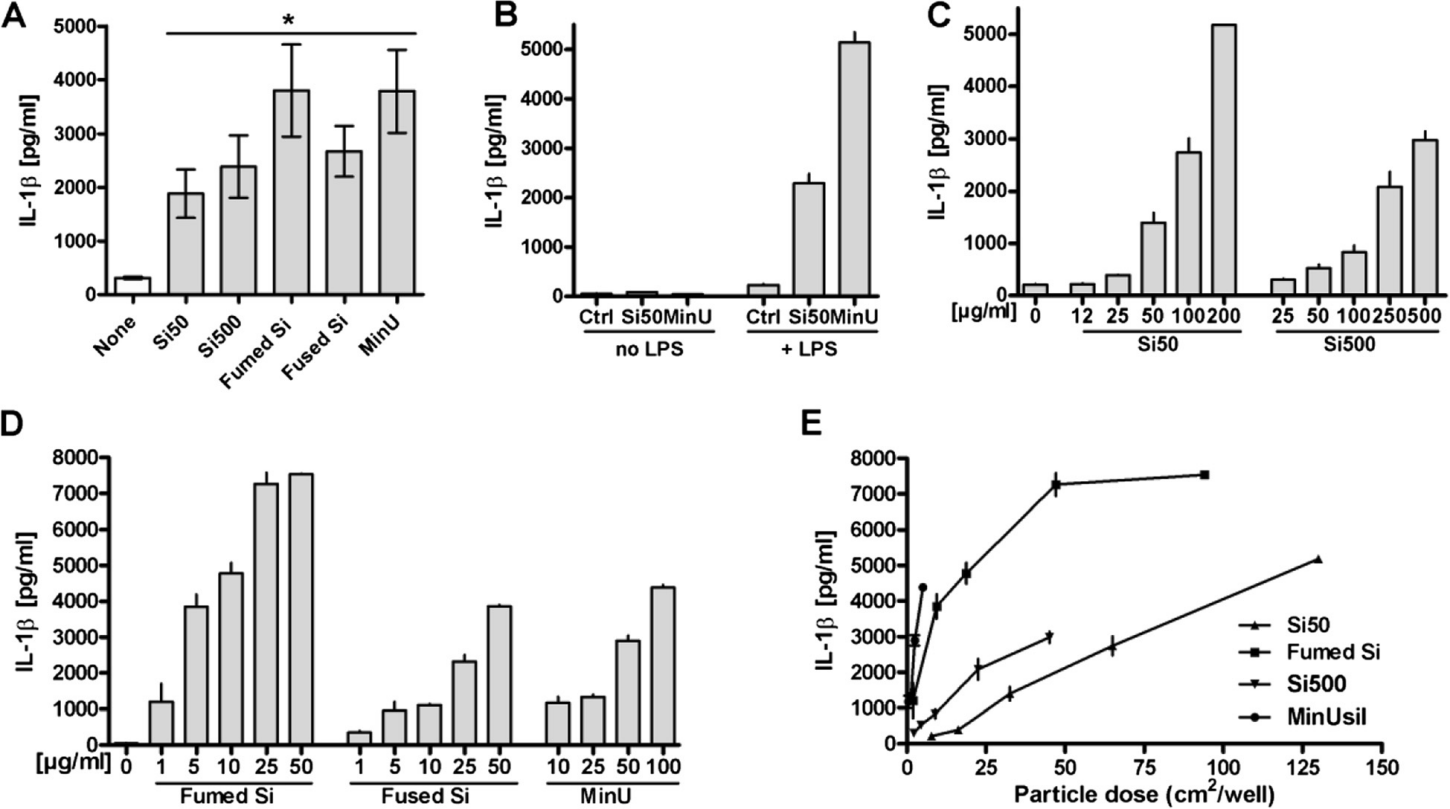
**IL-1β release induced by non-crystalline and crystalline silica particles in primary rat lung macrophages.** The cells were primed for 3 h with and without LPS (25 ng/ml) and subsequent exposed to different non-crystalline silica particles and MinUsil 5 (MinU) for 6 h. **A**) Effects of the 200 μg/ml Si50 and Si500, and 50 μg/ml fumed silica (fumed Si), fused silica (fused Si) and MinU in LPS-primed cells **B**) Effects with or without LPS and subsequent exposure to Si50 (200 μg/ml) and MinU (50 μg/ml) **C**) Concentration (mass/ml)-response relationships for Si50 versus Si500 on IL-1β release in LPS-primed cells **D**) Concentration (mass/ml)-response relationships for fumed- and fused silica and MinU on IL-1β release in LPS-primed cells. Fused silica particles after wet sedimentation were used. **E**) IL-1β responses in relation to particle surface areas in LPS-primed cells. In E the results for Si50, Si500 and fumed silica particles, besides MinUsil 5, are presented. The IL-1β levels were determined by ELISA. In **A**) the variability of the results is presented by the mean ± SD, including data from 5 experiments. * = p < 0.05 versus control, using Kruskal-Wallis test with Dunn’s Multiple Comparison test as a post-test. In **B- E** representative experiments are given, all performed in duplicates as indicated.

The effects of the different particles on IL-1β release were also examined in primary rat lung macrophages. All the particles induced significant IL-1β responses. In Figure 3A the variability of the IL-1β responses to 200 μg/ml Si50 and Si500, 50 μg/ml fumed silica, fused silica and MinUsil 5 in LPS-primed cells, is illustrated. In Figure 3B the responses to Si50 and MinUsil 5 with and without LPS-priming were compared, showing virtually no responses without LPS and large responses with LPS. In Figure 3C and D the concentration-response relationships to all the particles were compared, related to mass. Notably, the sensitivity of primary alveolar rat macrophages to all these silica particles was markedly greater compared to RAW264.7 macrophages. Furthermore, the magnitude of IL-1β release (fold-increase) was much greater in the primary cells than in the RAW264.7 macrophages. On a mass basis the nano-sized fumed silica particles were the most potent of the non-crystalline silica particles, with effects as low as from 1–5 μg/ml (equivalent to approximately 12.5-25 cm^2^/well). The submicro-sized fused silica particles also induced an IL-1β response at rather low concentration (from 5–50 μg/ml), but was markedly less potent than the fumed silica particles. The mono-disperse Si500 showed less potency than Si50 on a mass basis. MinUsil 5 induced marked IL-1β responses as low as 10 μg/ml. When comparing the IL-1β response in relation to particle surface area the order of potency was: MinUsil 5 > fumed silica > Si500 > Si50 (Figure 3E). For the MinUsil 5 (delivered by the same producer) the surface area obtained by Warheit et al. 2007 [30], (5,1 m^2^/g) was used in the calculation. The cytotoxicity, as assessed by LDH-release after 6 h exposure, was also examined. At 50–100 μg/ml fumed silica showed a 2-fold increase, whereas Si50 showed no increase at these concentrations. In comparison, the IL-1β release in the parallel experiments was many-fold larger (data not shown).

### Particle-induced IL-1β release via caspase-1 and NALP3 in RAW264.7 -macrophages

**Figure 4:**
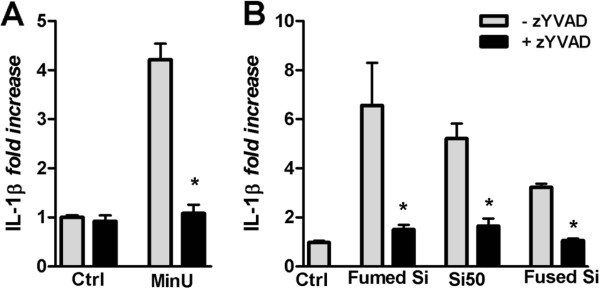
**Effect of caspase-1 inhibition on IL-1 β release in RAW 264.7 macrophages.** The cells were primed for 3 h by LPS (25 ng/ml) and subsequently exposed to silica particles after 6 h. The caspase-1 inhibitor zYVAD (10 μM) was added 1 h prior to exposure to MinUsil 5 (MinU, 200 μg/ml) (**A**) and 200 μg/ml Si50, fumed silica and fused silica (**B**), and the IL-1β responses were determined by ELISA. For fused silica, particles after wet sedimentation were used. In **A, B** the IL-1β levels represent 6 experiments, and are presented as mean ± SD of fold change. * = p < 0.05 significant different from particle-stimulated cells without zYVAD, using Kruskal-Wallis test with Dunn’s Multiple Comparison test as a post-test. **C**) Levels of pro-IL-1β. The cells were pre-treated with and without LPS for 3 h, and exposed to with Si50 (200 μg/ml) for 6 h and assessed for pro-IL-β levels by Western analysis. A representative Western blot is shown. In the lower part the band intensity of this experiment is shown.

**Figure 5:**
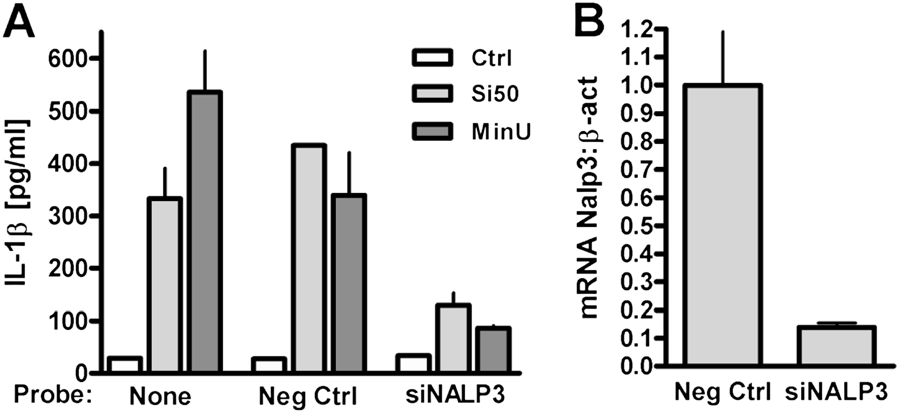
**Non-crystalline and crystalline silica particles induce IL-1β release in RAW264.7 macrophages via the NALP3 inflammasome. A**) The cells were transfected with siRNA against NALP3 as well a negative control for 48 h, then primed for 3 h by LPS (25 ng/ml before exposure to Si50 (200 μg/ml) or MinUSil 5 (200 μg/ml). The IL-1β levels were determined by ELISA. **B**) The NALP3 mRNA levels after transfection by siRNA against NALP3 and by a negative control siRNA. The NALP3 mRNA levels were adjusted against the respective actin-mRNA levels. Data presented are representative of two experiments performed in duplicates.

The roles of pro-IL-1β changes and caspase-1 and NALP3 in induction of IL-1β release by non-crystalline silica particles in RAW264.7 macrophages were examined. Figure 4A shows that MinUsil 5-induced IL-1β release was almost completely inhibited by the specific caspase-1 inhibitor, zYVAD (10 μM). Similarly, zYVAD completely inhibited the IL-1β release induced by the fumed silica particles, Si50 and fused silica particles (Figure 4B). The basal IL-1β levels were not affected by zYVAD. Figure 4C shows by Western blotting that LPS markedly increased the levels of pro-IL-1β in cell lysates, and that exposure to 200 μg/ml Si50 reduced the levels. To assess the involvement of NALP3, a siRNA gene silencing probe against the NALP3 receptor was used. As shown in Figure 5A, the NALP3 siRNA knockdown suppressed the IL-1β response to MinUsil 5 and Si50 in RAW264.7 macrophages. A similar suppressive effect was not found for the non-specific negative control siRNA. RT-qPCR was used to confirm approximately 85% NALP3 gene knock-down efficiency relative to cells transfected with the negative control probe (Figure 5B).

### Mechanisms in inflammasome activation by non-crystalline silica in RAW264.7 macrophages

**Figure 6:**
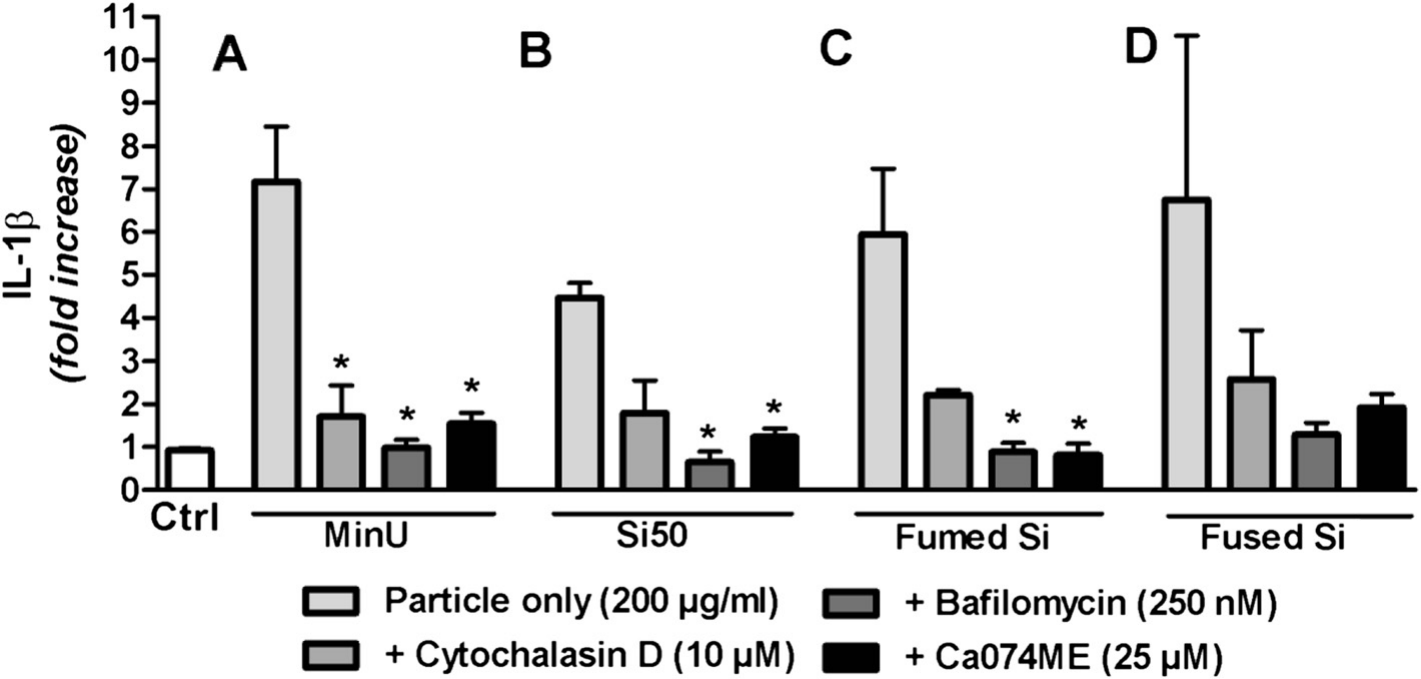
**Involvement of uptake and lysosomes in inflammasome activation by non-crystalline and crystalline silica in RAW264.7 macrophages.** The cells were primed for 3 h by LPS (25 ng/ml), and then pre-treated for 30 min with the indicated concentrations of cytochalasin D, bafilomycin or Ca074Me before exposure to different silica particles (200 μg/ml) for 6 h. **A**) MinUsil 5 (MinU); **B**) Si50 **C**) Fumed silica particles **D**) Fused silica particles. The IL-1β levels were determined by ELISA. Data represent the mean ± SD of 6 experiments and are presented as fold change. * = p < 0.05; significant different from particle-stimulated cells without inhibitors (light grey bars); using Kruskal-Wallis test with Dunn’s Multiple Comparison test as a post-test.

To examine the role of particle uptake and endosome membrane destabilization after phagocytosis, with subsequent leakage of the activated lysosome protease cathepsin B into cytosol, we used a panel of inhibitors [14]. The inhibitors cytochalasin D (inhibitor of actin assembly and phagocytosis), bafilomycin (inhibitor of vacuolar H + −ATPase and acidification of endosomes) and Ca074Me (inhibitor of cathepsin B) were added before exposure to the silica particles. The results showed that all inhibitors markedly reduced IL-1β release from RAW264.7 macrophages upon exposure to 200 μg/ml MinUsil 5, Si50, fumed silica and fused silica, although significance was not reached in all instances (Figure 6).

## Discussion

Crystalline structures such as quartz particles have previously been reported to activate the NALP3 inflammasome [14,25], a mechanism suspected to play an important role in the development of diseases such as silicosis [26]. In the present study we show how different non-crystalline (amorphous) silica particles from nano- to submicro-sizes are potent inducers of inflammasome activation, thus acting via the NALP3 assembly, leading to a caspase-1-dependent pro-IL-1β maturation and IL-1β release. When related to surface area the order of potency for IL-1β release in the lung macrophages was MinUsil 5 > fumed, silica > Si500 > Si50. The non-crystalline and crystalline silica particles are suggested to operate via similar mechanisms, since inhibitors of uptake and phagosomal stabilization seem to affect the inflammasome activation by these different particles in a similar manner, as demonstrated in RAW-macrophages.

Our results support and elaborate previous findings suggesting that a crystalline structure is not required for silica particles to activate the inflammasome. Non-crystalline silica particles have been found to induce a boost of IL-1β release in LPS-primed murine macrophages [29], TPA-primed THP-1 monocytic cells [27] murine dendritic cells [28] and keratinocytes [29]. In the dendritic cells the existence of an inflammasome mechanism was demonstrated by using cells from NALP3- and caspase-1-deficient mice [28]. For the THP-1 cells the involvement of an inflammasome mechanism was supported by inhibition of IL-1β release by zYVAD, an inhibitor of caspase-1 [27]. In the present study the inflammasome mechanism induced by different non-crystalline silica particles was demonstrated both by use of the caspase-1 inhibitor zYVAD and by siRNA against the NALP3 in RAW274.7 macrophages.

Some studies also point to other nanoparticles that may induce NALP3 inflammasome responses, such as TiO_2_-particles, amino-functionalized polystyrene particles and carbon black particles in macrophages [29,31-33]. In contrast, nanoparticles, like diesel exhaust particles, ZnO-NPs and carboxyl-functionalized polystyrene NPs [25,29,31], do not seem to show such responses. A remaining question is which nanoparticle characteristics and types of functionalisation are required to elicit NALP3 inflammasome responses.

In previous studies the IL-1β responses to non-crystalline particles varied with size, and also with cell type used. Morishige and coworkers [27] observed that the non-crystalline silica particles of 1000 nm size induced an IL-1β release in TPA-primed THP-1 cells, whereas the 30 to 300 nm counterparts were without effect. In murine macrophages approximate similar IL-1β levels were reported in the presence of a high concentration of non-crystalline silica particles of nano (15 nm)- and micrometer (1.5 μm)-size, whereas only the nano-sized particles induced IL-1β release in human keratinocytes [29]. Winter and coworkers [28], showed that 14 nm silica nanoparticles induced a marked IL-1β release in LPS-primed murine dendritic cells. In the RAW264.7 macrophages and rat primary lung macrophages we observed that both silica particles from the nano-size (Si50, fumed silica) and submicro-size (Si500 and fused silica), besides the crystalline MinUSil 5, induced marked IL-1β responses.

The potential to induce inflammatory responses at low concentrations is a critical question. In dendritic cells, the potency of the non-crystalline silica nanoparticles (14 nm) on a mass basis was approximately similar to quartz (DQ12) [28]. In our study non-crystalline Si50 was less potent than quartz (MinUsil 5) on a mass basis, when examined in primary lung macrophages. The fumed silica with the smallest size (5–20 nm), but also a different composition, was however on mass basis the most potent of all the particles examined, giving IL-1β responses from rather low concentrations (1–5 μg/ml). This supports the potential relevance of these findings, suggesting that additional toxic studies on these particles could be of interest. In other studies the concentrations of non-crystalline silica particles used to induce IL-1β release were mostly in a higher range [27,28], than those used in the present study.

In general, in many previous studies using cell cultures, nano-sized particles seemed more potent compared to larger particles of the same composition, when presented on a mass basis. However, these differences tend to disappear when relating the responses to particle surface area both upon *in vitro* and *in vivo*[34,35] exposure. In accordance with this, we find that when assessing the IL-6 and IL-8 response to Si50 and Si500 in BEAS-2 B-cells, the particle surface area seems to be the critical determinant (unpublished results). However, with regard to the IL-1β responses in primary rat lung macrophages and in particular in mouse RAW-macrophages, the Si500 silica particles seemed markedly more potent than Si50 of the same composition, when presenting the responses in relation to particle surface area. These somewhat surprising finding could be due to a different role of particle uptake for various cytokine responses. Our previous study indicated that the increased IL-6 and IL-8 release following Si50 in BEAS-2 cells seemed to occur independently of particle uptake [36]. In contrast, the IL-1β response in RAW264.7 macrophages, shown in the present study, seemed to be dependent on uptake of the silica particles (see below). Notably, it is known that particle uptake via phagocytosis is less effective with nano-sized particles than with larger particles around 0.5 μm [37]. Thus, the present finding with Si500 as more effective inducers of IL-1β than Si50 in RAW264.7 macrophages and primary rat lung macrophages at comparable particle surface areas could be related to more effective particle uptake, although it cannot be excluded that other explanations may be involved. Previous inhibitor studies have shown that uptake of micro-sized crystalline silica particles in alveolar macrophages occurring via actin-dependent phagocytosis [38] is required for eliciting inflammasome response [14]. In our study, uptake of all the non-crystalline silica particles of various sizes also seemed to be mediated via a mechanism that was actin-dependent, as cytochalasin D inhibited the IL-1β responses. However, both phagocytosis and macropinocytosis are actin-based pathways [37]. For nano- and submicro-sized particles, that are relevant for the present study, it has been reported that the extent of uptake in macrophages differ, with several mechanisms operating for particles of different sizes and functionalisation [37,39-43]. Furthermore, the mechanisms of uptake are dependent on the differentiation state of the macrophages and the proteins that the nanoparticles encounter in culture media. Thus, in primary macrophages opsonised by serum proteins nanoparticles (polystyrene) were taken up by phagocytosis, whereas other mechanisms operated in monocytic THP1 cells with and without differentiation with TPA [42]. The similar patterns of responses to cytochalasin D and the other inhibitors to all the different particles observed in the present study were surprising, as it points to a common mechanism operating over the whole particle size range. One possible explanation could be that the secretion of IL-1β from the cells was actin-dependent. Previous studies in bone marrow dendritic cells have however shown that cytochalasin D does not interfere with the secretion process of IL-1β [14]. More conceivably, the agglomeration state of the particles could be of importance for the similar responses of nano-and submicro-sized silica particles to cytochalasin D. Thus, the macrophages might sense all the different particles as sub-micrometer agglomerates, pointing to phagocytosis as a common mechanism for particle uptake. This was discussed by Winter et al. [28], but the agglomeration state of the non-crystalline particles was not assessed. In the present study the hydrodynamic size of all the particles in the culture medium with serum, was from 90 nm and larger. The optimal particle size for phagocytosis has been reported to be relative large, 250 nm to 3 μm, whereas nanoparticles less than 250 nm were less effectively phagocytosised [37,41]. Further studies are, however, required to approach the importance of the agglomeration process for the inflammasome activation.

With further respect to mechanisms, our findings suggest that the IL-1β responses induced by the non-crystalline particles in the RAW264.7 macrophages involve activation of the vacuolar H^+^-ATPase, and induction of cathepsin B release, subsequently leading to phagosomal destabilization, as previously reported for quartz particles [14]. In a recent study, Morishige [27] found that the IL-1β response induced by 1000 nm non-crystalline silica particles was markedly reduced by inhibitors of these processes. Here, we report that the IL-1β responses by all the different silica particles were nearly abolished upon pre-treating the RAW264.7 macrophages with relevant inhibitors such as bafilomycin A1 and CA-074-Me. Thus, our findings corroborate that non-crystalline silica particles of different sizes and composition and crystalline silica particles, act via similar mechanisms. Previous studies in endothelial cells have shown that non-crystalline silica particles may induce cytotoxic responses, with the particles in nano-size as most potent on a mass basis [44]. Potentially, such cytotoxicity could influence the silica-induced IL-1β responses in our mechanistic studies performed at higher concentrations, in which different inhibitors were used. At the early time points (6 h) used to assess the IL-1β responses, however, the particles induced little toxicity as assessed by LDH-release. In comparison the IL-1β release was much larger, thus strongly arguing that the observed responses are not due to release of pro-IL-1β as a result of plasma membrane rupture. This is also contradicted by the fact that zYVAD and siRNA against NALP3 inhibit the SiNP-induced IL-1β responses.

The main goal in the present study was to investigate the ability of non-crystalline particles to act via inflammasome activation in macrophages. We have also compared the relative potency of the non-crystalline silicas to crystalline silica (MinUsil 5) with respect to IL-1β release in the primary rat lung macrophages. When related to surface area the crystalline silica was more potent than the non-crystalline silicas (in particular compared to Si50 and Si500), which could suggest a role of crystallinity. However, the findings showing that fumed (non-crystalline) silica particles induced almost the same response as crystalline silica, suggest that other factors/mechanisms also may contribute. Thus, the overall response pattern obtained may be influenced by differential uptake mechanisms for the different silica particles in the lung macrophages (as previously discussed), but also to other differences in particle surface reactivity than caused by crystallinity. Notably, studies with different quartz particles (nanoscale- and fine quartz) by Warheit and coworkers [30] indicate that the toxicity of different particles is more dependent upon particle surface activity effects than particle size and surface area.

Based on these studies it would be interesting to explore any possible acute inflammatory effects of non-crystalline silica particles, in particular for relevant concentrations of nano-sized particles in LPS-primed animals. Any possible implications for possible long-term effects are not likely as no or minor effects from long-term industrial use of non-crystalline (amorphous) silica particles have been reported. This is probably due to the reduced retention time of the non-crystalline silica particles in the lung tissue compared to quartz.

## Conclusions

In conclusion, different non-crystalline silica particles from nano- to submicro-metre size ranges induced IL-1β release from LPS-primed RAW264.7 macrophages and primary rat lung macrophages. In the primary rat lung macrophages the silica particle-induced IL-1β responses occurred at low concentrations, being more sensitive as a model system, than the RAW264.7 macrophage cell line. In the lung macrophages crystalline silica (MinUsil 5) was more potent in inducing IL-1β release than the non-crystalline silica particles, but much less versus fumed silica particles than versus Si50 and Si500, when adjusted to surface area. The differential response patterns obtained suggest that silica-induced IL-1β responses not only depend on the particle surface area, but on factors and/or mechanisms such as particle reactivity or particle uptake. The studies in RAW264.7 macrophages showed that silica-induced IL-1β responses occurred via mechanisms involving particle uptake, phagosomal destabilization and NALP3 inflammasome activation. These findings may indicate that bacterial infection via PAMPs may augment acute inflammatory effects of different types of non- crystalline and crystalline silica particles, and thus their acute health hazard potential.

## Methods

### Sample characterisation

Two different mono-disperse non-crystalline (amorphous) silica particles (provided by Kisker Biotech GmbH & Co, Steinfurt; Germany) with a nominal size of 50 nm (Si50) and 500 nm (Si500) were used. In addition, two types of poly-disperse, non-crystalline silica particles were included in this study; fumed silica (Aerosil 200, Degussa Evonik, Germany) and fused silica (Suprasil, Heraeus, Germany). MinUsil α-quartz particles (crystalline silica, MinU-sil 5 from U.S. Silica Co., Berkeley Springs, WV, US, were also used. The Si50 and Si500 particles were prepared from an analytically pure silane precursor via a sol–gel process and supplied in de-ionized, sterile-filtered water. This method is known to result in 100% pure amorphous silica particles. Both fumed silica and fused silica are prepared from chemically pure chlorosilane precursors via a pyrogenic process. Fumed silica is collected as fluffy white powder with a primary particle size of 5–20 nm, whereas fused silica is delivered in the form of massive glass rods. The glass rods were crushed and milled in a swing mill in order to produce sufficiently small particles of angular morphology. The amorphous character was confirmed by X-ray diffraction (AXS D8 Advance, LynxEye silicon strip detector). No traces of crystalline silica polymorphs were detected. For the fused silica a fraction with the smallest particles obtained after wet sedimentation was used for determination of size by dynamic light scattering (DLS), ζ-potential, and in assessing cellular responses.

Stock solutions were prepared in sterile water (final concentration 2 mg/ml) with bovine serum albumin (BSA, final concentration 1.5%), and phosphate-buffered saline (PBS, final dilution 1x) added after sonication, as according to Bihary et al. [45]. The non-crystalline silica particles were analyzed for their morphology/dimension/surface charge and area. The morphology of the non-crystalline silica particles was assessed by transmission electron microscopy (TEM), using a JEOL JEM 1220 (mono-disperse silicas) and a Philips CM 30 (fumed and fused silicas). The specific surface areas of the mono-disperse particles were determined by the single-point Brunauer, Emmet and Teller (BET) procedure on a customized apparatus developed according to literature specification (J.M Thomas; W.J.Thomas, 1997) after a slow evaporation at room temperature of water from the particle suspensions. The BET surface areas of fumed and fused silica were determined with a Micromeritics Tristar instrument. The hydrodynamic diameters of the silica particles were determined in water and in culture media with 10% fetal calf serum (FCS) by DLS (Malvern Instruments LTD, UK), instantly and 2 h after dispersion of the particles in the medium. The ζ-potentials of the silica particles were determined in water, using a 100 μg/ml final concentration in a nano-sizer (Malvern instruments, UK).

### Cell cultures

The mouse macrophage RAW264.7 cell line was purchased from American Type Tissue Culture Collection (ATCC, Rockville, MD) and maintained according to ATCC protocols. Briefly, RAW264.7 cells were cultured in DMEM with 10% FCS, penicillin-streptomycin and 2 mmol/L L-glutamine (Gibco BRL; Paisley, Scotland, UK) in 12-well plates (Corning, Lowell, MA, USA), with a density of 3x10^5^ cells/well, one day prior to exposure, The rat primary lung macrophages were isolated and cultured according to a procedure previously described [46]. Briefly, the macrophages obtained by lung lavage of male rats (WKY/NHsd, purchased from Harlan, UK), were suspended and cultured in RPMI-medium, with antibiotics and 5% fetal bovine serum (FBS). Non-attached cells were removed after 1 h in culture, and fresh RPMI-medium with FBS was added to the attached cells. The purity of the macrophages exceeded 90%. Both the RAW264.7 macrophages and the primary rat lung cells were pre-treated with or without lipopolysaccharide (LPS, 25 ng/ml, Sigma-Aldrich Chemical Company, St Louis, MO, USA) for 3 h, before exposure to increasing concentrations of the respective particles for 6 h.

In some experiments, an inhibitor of actin assembly (cytochalasin D, 10 μM Sigma-Aldrich Chemical Company, St Louis, MO, USA), an inhibitor of caspase-1 activity (zYVAD-fmk, 10 μM, EMD Chemicals Inc, Gibbstown, USA), an inhibitor of vacuolar H^+^-ATPase (bafilomycin A1, 250 nM, Calbiochem, Merck KGaA, Darmstadt, Germany) and an inhibitor of cathepsin B (Ca-074ME, 50 μM, Sigma-Aldrich Chemical Company, St Louis, MO, USA) were added to the cells 30 min prior to silica particle exposure. RAW264.7 macrophages have previously been reported not to have any NALP3-inflammasome response, due to a lack of ASC [47]. Here, however, we observed solid expression of ASC in the RAW264.7 macrophages used, as measured at the mRNA-level (data not shown); and similar IL-1 β responses were observed in LPS-primed J774A.1 macrophages, a cell line known to be sensitive with respect to inflammasome activation [47] (data not shown).

### Transfection by siRNA

Pre-designed siRNA probes against NALP3 (siRNA ID: s103711), and a certified non-silencing control (Silencer® siRNA Starter kit) were purchased from Applied Biosystems, Life Technologies Corporation, CA USA. RAW264.7 macrophages were plated in 12-well plates as described above and transfected using HiPerfect reagent (Qiagen, GmbH, Hilden, Germany) and 5 nM siRNA. The transfection reagent and the siRNA probes were mixed by vortexing in OptiMEM (Invitrogen, Life Technologies Ltd, UK) and incubated for 10 min in room temperature before the complex was added drop-wise into the cells suspension. Cells were then incubated for 48 h with transfection complexes in normal medium, washed and incubated for additional 48 h in medium only. The cells were thereafter exposed to LPS (25 ng/ml) and silica particles (200 μg/ml) for up to 9 h before harvesting and subsequent RT-PCR analyses of the silencing.

### Cell viability

The toxicity of different silica particles and siRNA transfection was assessed investigating cell membrane integrity using a lactate dehydrogenase (LDH) leakage assays (“LDH detection kit”, Roche Applied Biosystems, Penzberg, Germany).

### Enzyme immunoassays (EIA)

Cell culture media were collected and centrifuged at 300 x g to remove cell debris and at 8000 x g to remove suspended silica particles. The final supernatants were stored at −70°C until cytokine analysis. The amount of IL-1β in cell medium was measured by ELISA (R&D systems, Minneapolis MN, USA) according to the manufacturers’ guideline. An increase in color intensity was quantified by a plate reader (TECAN Sunrise, Phoenix Research Products, Hayward, CA, USA) equipped with a dedicated software (Magellan V I.10).

### Real time quantitative RT-PCR

Quantification of mRNA was performed using ABI Prism Fast Real time PCR System (Applied Biosystems, Foster City, CA). Primers for NALP3 (forward primer: 5’- TCGACCCTTGGACCAGGTTCAGT -3’, reverse primer: 5’- CATGCCCGGGTCTCCCAGAGT -3’), β-actin (forward primer: 5’-GCAGCTCCTTCGTTGCCGGT-3’, reverse primer: 5’-TACAGCCCGGGGAGCATCGT-3’) and for ASC (forward primer: 5’-TGGCTGAGCAGCTGCAAACGA-3’, reverse primer: 5’-TGCTGGTCCACAAAGTGTCCTGTT-3’), were designed using the NCBI primer-BLAST software (http://www.ncbi.nlm.nih.gov/tools/primer-blast/). One pair of ASC primers was adapted from Pelegrin [47]. Cell harvesting and RNA preparation were performed according to the “Cells-to-Ct” Kit protocol (Applied Biosystems, Life Technologies Corporation, CA USA) and qPCR was performed by using Master Mix for Power SyBr Green and 300 nM primers. Gene expression of the house-keeping gene β-actin was used for normalization.

### Western blotting

The pro-IL-1β levels were also quantified by Western analysis. After exposure to particles, the cell-culture dishes were washed twice with PBS, before the dishes were stored at - 70°C until further processing. Frozen cell culture dishes were put on ice and 200 μl lysis buffer was added. After 5–10 min the cells were scraped and sonicated prior to protein determination, using the BioRad DC Protein Assay (BioRAD Life Science, CA, USA). The samples (70 μg protein/well) were run on SDS-PAGE (15%) gels before transferring onto nitrocellulose membranes (NEN, Life Science, Boston, MA, USA). To ensure that the protein levels of each well were equal, Ponceau-staining was used. After blocking, membranes were incubated with rat antibody against IL-1β (Santa Cruz, CA, USA), prior to exposure with species-specific horseradish peroxidase-coupled secondary antibodies (Cell signaling, Beverly, MA, USA). The blots were developed using the Super-Signal West Dura chemoluminiscience system (Pierce, Perbio Science, Sweden) according to the manufacturers’ instructions. Finally, the membranes were stripped by incubations for 15 min at room temperature with mild antibody stripping solution, and re-probed with mouse anti-β-actin (Sigma-Aldrich; St. Louis; MO, USA).

### Statistical analysis

Data are presented as mean ± standard deviation (SD). Probability values were considered significant at p <0.05. The data were analysed by the nonparametric one-way ANOVA analysis; Kruskal-Wallis test. Dunn’s Multiple Comparison test was used as a post-test, when comparing samples inside the dataset.

## Competing interests

The authors declare that they have no competing interests.

## Authors’ contributions

WJS was responsible for the “research idea”, performed most of the experimental work and drafted a major part of the manuscript. ML and JH were involved in the planning, running interpretation of the experiments, and drafting of the manuscript. BF was responsible for providing and characterization of the fumed and fused silica samples, and in drafting parts of the manuscript. MG was involved in the initial experiments on inflammasome activation, besides TEM and surface area measurements of Si50 and Si500, and also helped in drafting of the manuscript. MK was involved in the drafting of the manuscript. PES was involved in the planning of the study, and also the drafting of the manuscript. TS was performing the analysis with DLS characteristics of all the particles, the experiments with Westerns, and also involved in the drafting of the manuscript. MR was together with WJS responsible for the experimental design, the planning and running of the experiments and the drafting of the manuscript. All authors read, commented and approved the manuscript.

## Supplementary Material

Additional file 1 **Figure S1.** Hydrodynamic sizes of Si50 and Si500 particles in water and in culture medium. **Figure S2.** Hydrodynamic sizes of fumed and fused silica particles in water and in culture medium. Click here for file
