# Supplementary material for: Comparison of non-crystalline silica nanoparticles in IL-1β release from macrophages
Source: Part Fibre Toxicol. 2012 Aug 10;9:32. doi: 10.1186/1743-8977-9-32 (PMC3441334; doi:10.1186/1743-8977-9-32)
Supplement: Additional file 1 — Figure S1. Hydrodynamic sizes of Si50 and Si500 particles in water and in culture medium. Figure S2. Hydrodynamic sizes of fumed and fused silica particles in water and in culture medium. [file 1743-8977-9-32-S1.pdf]

Additional file 1

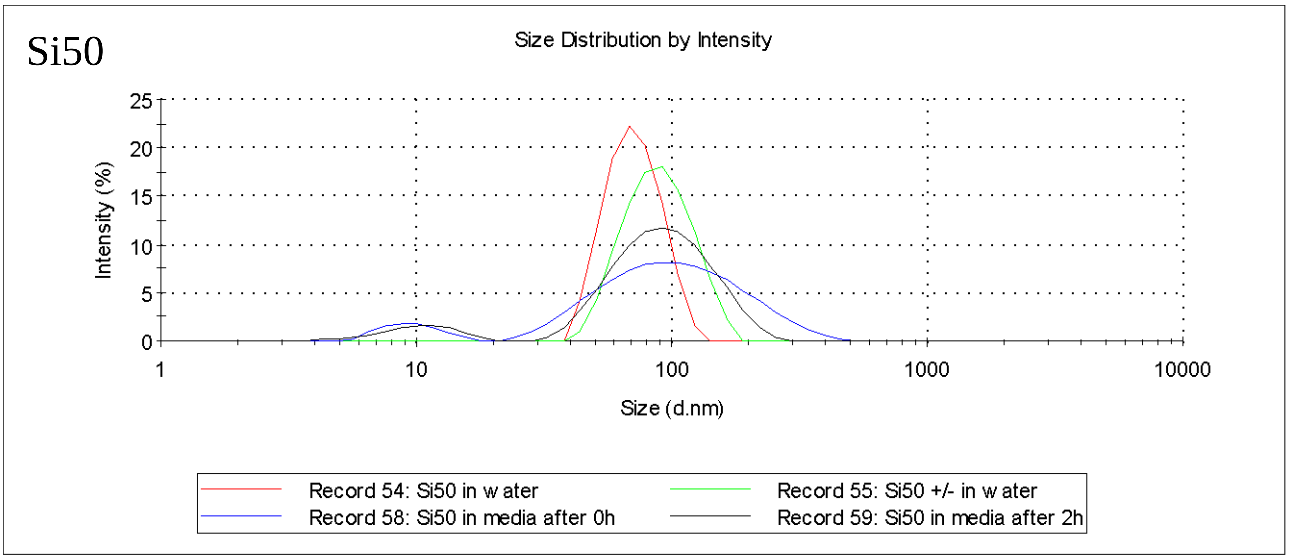

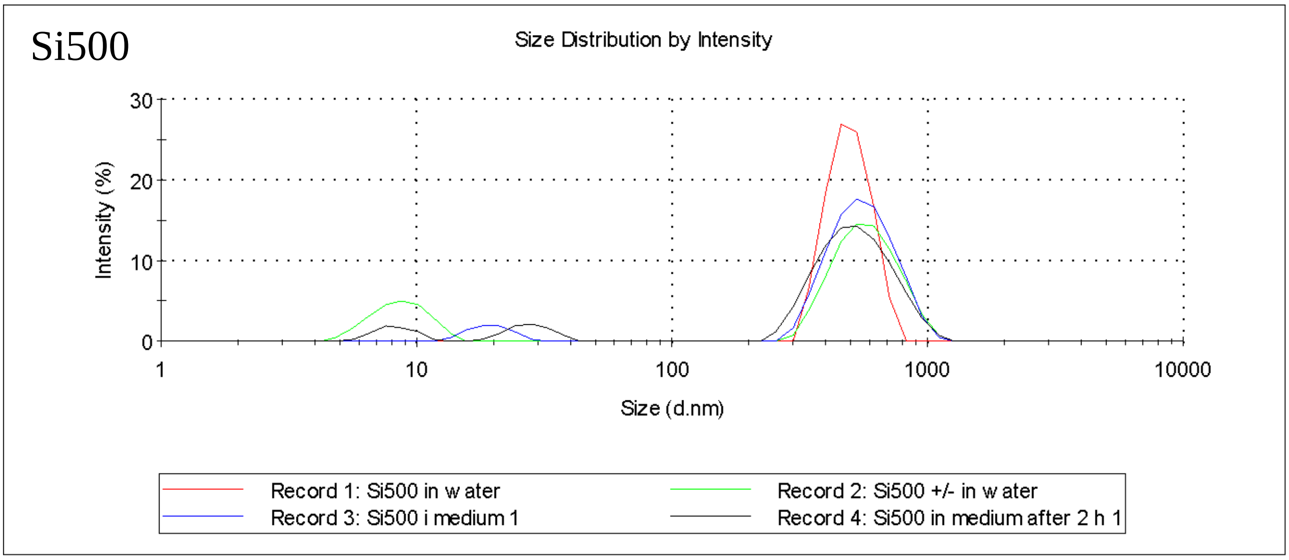

Figure 1. Hydrodynamic sizes of Si50 and Si500 particles in water and in culture medium.

The hydrodynamic sizes of the different particles were determined by dynamic light scattering (DLS). The particles (100 µg/ml) were dissolved in sterile water (red line), and in sterile water with BSA and PBS added after sonication (+/- green line), as described in Materials and Methods. The particles (100 µg/ml) supplemented with BSA/ PBS were also dispersed in DMEM culture medium plus 10% FCS, and instantly monitored by DLS (blue line) and subsequently after 2 h (black line). The figure is representative for two experiments.

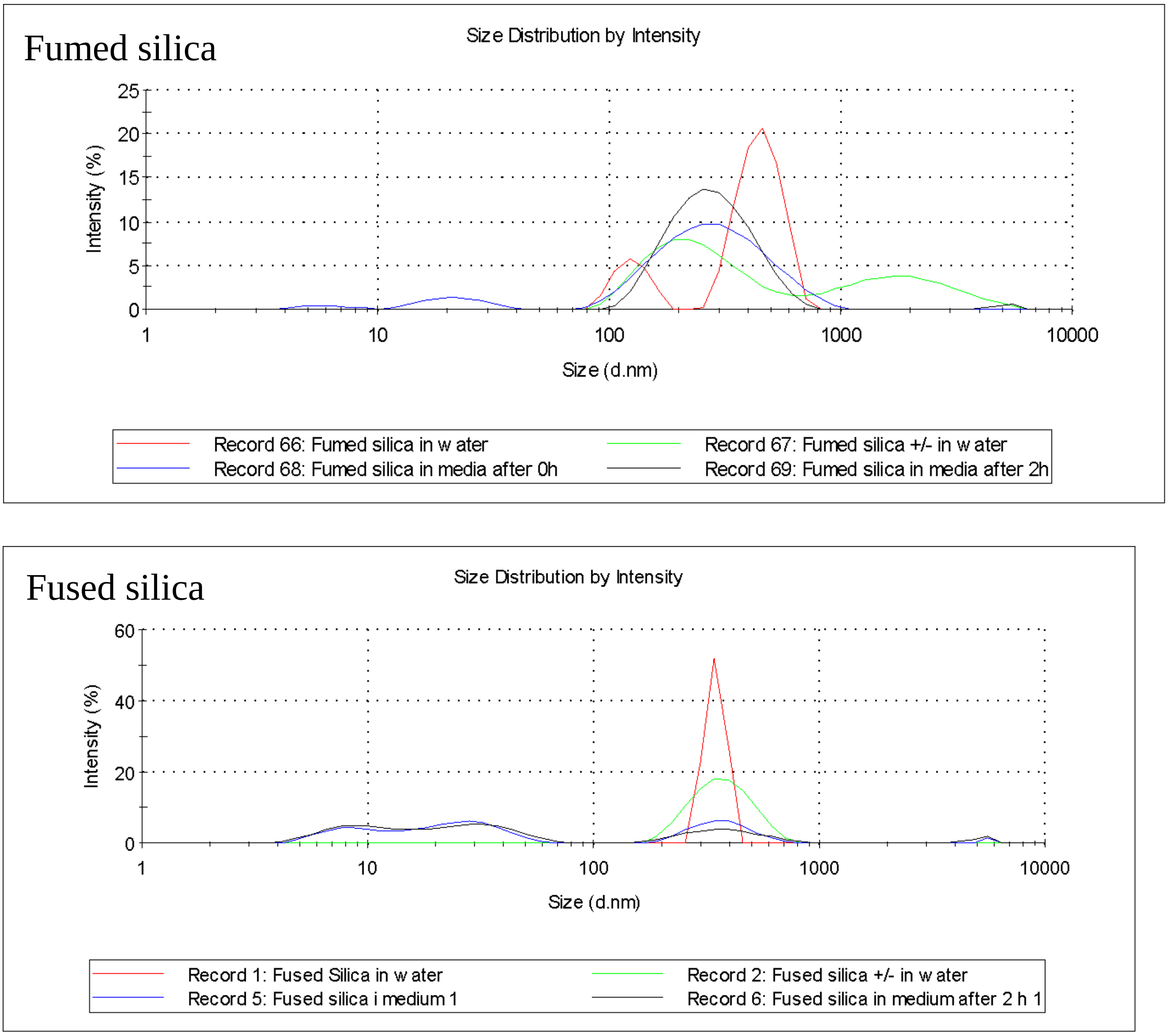

Figure 2. Hydrodynamic sizes of fumed and fused silica particles in water and in culture medium.

The hydrodynamic sizes of the different particles were determined by dynamic light scattering (DLS). The particles (100 µg/ml) were dissolved in sterile water (red line), and in sterile water with BSA and PBS added after sonication (+/- green line), as described in Materials and Methods. The particles (100 µg/ml) supplemented with BSA/ PBS were also dispersed in DMEM culture medium plus 10% FCS, and instantly monitored by DLS (blue line) and subsequently after 2 h (black line). The fused particles only include the smallest fraction after wet sedimentation as depicted by ** in Table 1. The figure is representative for two experiments.
